# Technical quality of root canal fillings done in a Nigerian general dental clinic

**DOI:** 10.1186/1472-6831-12-42

**Published:** 2012-10-15

**Authors:** Ezekiel Taiwo Adebayo, Lilian Ejije Ahaji, Rita Nneka Nnachetta, Olaitan Nwankwo, Nonye Akabogu-Okpeseyi, Morufu Olasunkanmi Yaya, Nurudeen Ayoola Hussain

**Affiliations:** 1 Army Dental Centre, Military Hospital, General Post Office Box 3338, Kaduna, Nigeria; 2 Department of Public Health, Military Hospital, Awolowo Road, Ikoyi, Lagos, Nigeria

## Abstract

**Background:**

Previous reports indicate that worldwide, the technical quality of root canal fillings is poor. There are few reports from sub-Saharan Africa and none yet from Nigeria where most patients access treatment from non-specialists especially at general dental clinics. The aim of this study was to evaluate the technical quality of root canal fillings done in a general dental clinic with emphasis on the effects of professional experience of the operator, whether tooth was anterior or posterior and whether it was a maxillary or mandibular tooth.

**Methods:**

Retrospective study of case notes and periapical radiographs of patients with completed root canal fillings seen between 2008 and 2011. Inclusion criteria included cases of primary treatment with available case notes, good quality pre-operative and post-operative periapical radiographs. Technical quality that was assessed was root canal length and homogeneity. Root canal fillings were classified either as Good Quality Endodontic Work (GQEW) or Non- Good Quality Endodontic Work (NGQEW).

**Results:**

Fifty-one patients aged between 8 and 54 years (mean 28) fulfilled the inclusion criteria for this study. From these, there were 62 root filled teeth giving a ratio of 1.2 root canal filled teeth per person. There were acceptable length of root canal fillings in 71% of teeth, 58.1% were homogeneous while 53.2% were GQEW. There was no statistically significant difference in whether tooth was root filled by junior or senior dentist (p = 0.43), anterior or posterior (p = 0.11). There was significant association between GQEW and maxillary teeth (p = 0.03).

**Conclusion:**

This study showed that the overall technical quality of root canal fillings done by non-specialists was better than earlier reports but lower than that done by endodontists. Since many patients receive treatment from non-specialists in developing countries, it is necessary to improve technical quality of root canal fillings done in general dental clinics. These could be through improvement in the quality of undergraduate training and more post graduate continuing education courses for skills update.

## Background

Root canal therapy is widely recognized as an intricate dental procedure. Previous reports indicate that the therapy is often of poor quality [1-3]. The biological and therapeutic aim of root canal treatment is to prevent apical periodontitis or to create optimal conditions for healing of the periapical tissues. Removal of infection and the elimination of bacteria from the root canal system will help avoid re-infection [4].

Failed root canal fillings are associated with inadequate treatment, through either technical error or insurmountable difficulty in the canal system of the tooth [4,5]. Some of the factors that determine the technical quality are length and quality of the filling and coronal restorations [6-8]. The quality of root canal filings is assessed radiographically. Also, the availability and reliability of radiographs makes them suitable for epidemiological studies [9].

In developing countries such as sub-Saharan Africa, most patients receive treatment from non-specialist dental clinics due to paucity of endodontists. Among dental procedures performed are root canal fillings. Assessment of the quality of root canal fillings is important for purpose of evaluation but it is plagued by differences in criteria. In sub-Saharan Africa, studies of the quality of root canal treatments are rare. A previous report recorded that only 17.7% of root canal fillings in a Senegalese population were of good technical quality [10]. The aim of this study was to determine the technical quality of root canal fillings in a Nigerian general dental clinic with analyses of the effects of length of professional experience of the dentist, location of the tooth in the jaw and whether the root filled tooth was maxillary or mandibular.

## Methods

A retrospective review of case notes of all patients seen at the Army Dental Centre, Military Hospital, Ikoyi, Lagos, Nigeria between 2008 and 2011 was undertaken. Of these the following were selected out; those who completed root canal treatment, with adequate quality periapical radiographs taken before and after treatment. Those who had surgical procedures like cyst enucleation or apicectomy were excluded. Root canal therapy was by step-back technique with obturation using lateral condensation of cold gutta percha. The technical quality of the root canal fillings were reviewed by checking the post-treatment radiographs. All radiographs were reviewed by one dental surgeon. Intra-observer reliability for the reviewer was calculated by test-retest method, Cohen’s Kappa was 0.82. The study was approved by the hospital ethical committee. All patients consented to initial treatment and the collected data was only available for this study.

Technical quality of root canal fillings was evaluated based on adequacy and homogeneity using the criteria of Unal et al., [11]. Adequacy of root canal fillings were assessed as:

a. Root filling terminating 0-2 mm from the radiographic apex (acceptable).

b. Root filling terminating ≥2 mm from the radiographic apex (unacceptable).

c. Root filling extending beyond the radiographic apex (unacceptable).

Homogeneity of root filling was considered as:

a. Homogeneous root filling, good condensation, no voids visible (acceptable).

b. Non homogeneous root filling, poor condensation or voids present (unacceptable).

A tooth was considered to have good quality endodontic work (GQEW) if all root canals were of acceptable root filling length and homogeneous according to the criteria of Unal et al., [11].

Incisors and canine teeth were classified as anterior, while premolars and molar were regarded as posterior teeth. All the practitioners had no post-qualification endodontic training. The dentists of 0-5 years post-qualification experience were categorized as junior doctors while those with more than 5 years experience were categorized as senior.

Statistical analysis of the data was carried out using SPSS 10.0 for Windows (SPSS Inc, Chicago, IL, USA). Chi-square analysis of association was done at ≤0.05 level of significance.

## Results

Fifty-one patients met the study criteria. They were aged between 8 to 54 years (mean 28 ± 9.3 years). There were slightly more males (n = 28, 54.9%) than females (n = 23, 45.1%). From the 51 patients, 62 teeth were root filled giving a ratio of 1.2 teeth per person. Forty-four patients (86.2%) had one tooth root filled, 6 (11.8%) had two teeth while one (2%) had four teeth filled. Treatment was carried out between 1 to 8 visits (median 3). Radiographic assessment showed that root canal filling length was acceptable in 44 teeth (71.0%), while the rest were unacceptable because they terminated ≥2 mm from the radiographic apex (n = 13, 21.0%) and were overfilled (n = 5, 8.0%). More teeth had homogeneous root fillings (n = 36, 58.1%) than were unacceptable (non-homogeneous). Based on the criteria of Unal et al., [11], 33 teeth (53.2%) had GQEW while 29, 46.8% had Non Good Quality Endodontic Work (NGQEW). Table 1 showed that there was no statistically significant relationship between duration of professional seniority and the quality of endodontic work (chi-square =0.89, p = 0.34). In Table 2, there was no statistically significantly relationship between whether the tooth was anterior or posterior and the quality of endodontic work (chi-square = 2.51, p = 0.11). With Yates correction, the quality of endodontic treatment was statistically significantly associated with whether a tooth was in the upper or lower jaw. Teeth in the upper jaw were more likely to have GQEW while those in the lower teeth are NGQEW in Table 2 (Yates Corrected chi-square = 5.37, p = 0.03).

**Table 1:** Analysis of the quality of root fillings done by various categories of doctors at a Nigerian dental clinic

	**Senior doctor**	**Junior doctor**	**Total**
GQEW	12	21	33
NGQEW	14	15	29
TOTAL	26	36	62

Chi-square = 0.89, p = 0.34 GQEW- Good Quality Endodontic Work.

NGQEW- Non Good Quality Endodontic Work.

**Table 2:** Analysis of the relationship between the technical quality of root fillings, position and location of tooth in the jaws

	**GQEW**	**NGQEW**	**TOTAL**
**Position of teeth (Chi square = 2.51, P = 0.11)**			
Anterior	18	15	33
Posterior	10	19	29
Total	28	34	62
**Location of teeth (Yates Correction of Chi-square = 5.37 P = 0.03)**			
Maxillary	26	3	29
Mandibular	21	12	33
Total	47	15	62

GQEW - Good Quality Endodontic Work NGQEW- Non Good Quality Endodontic Work.

## Discussion

Assessment of root canal fillings is mostly by means of panoramic or periapical radiographs. According to Grőndahl et al., [12] and De Cleen et al., [13], panoramic radiographs have less inter-examiner reliability and readability. Our study used periapical radiographs and case notes for assessment of technical quality of root canal treatments. This was also the technique used by Unal et al., [11]. Different criteria have been used by workers to assess the acceptability of root canal fillings. Length of the root filling alone was used by others [13-16]. Some workers used length and homogeneity of the root canal filling [11,17-23]. However, this present study evaluated the quality of root filled teeth based on length and homogeneity. There is need to standardize the assessment of the quality of root canal fillings preferably using the guidelines of European Society of Endodontology [24] to enhance comparability.

Peak et al., [25] studied the outcome of root canal filings done by military general dentists in the Royal British Army. While 57% of the root fillings were definitely successful, the rest were probably successful (28%) and failures (15%). The study among the French revealed a lower quality as only 21% of root fillings were reported to be technically acceptable by Boucher et al., [26]. In a Taiwanese population-based study, Chueh et al., [27] found that root canals with adequate length of fillings were 61.7% while unacceptable fillings due to inadequate length were 25.0% and overfilling were 12.6%; 0.6% had no fillings in their root canals. As for homogeneous obturation, 38.0% had acceptable obturation. Our result was acceptable length of root canal filling in 74.2% of teeth. We also found that 58.1% of the teeth had homogeneous root fillings. Our results of length of root filling and obturation were better than the technical quality of the studies cited above.

In a meta-analytic study, Kojima et al., [28], reported that with respect to length of root fillings, there was significant difference in success rate between flush and over-extended and between flush and under-extended. They concluded that the root canal filling should be within 2 mm of the radiographic apex. Table 1 showed that professional experience gathered over long years of general dental practice may not necessarily improve the technical quality of endodontic work. There was no statistically significant difference in the quality of endodontic work done by either junior or senior dental surgeons. Table 1 showed that while there were more GQEW (n = 33, 53.2%) than NGQEW (n = 29, 46.8%), this clinical difference was not likely statistically significant. Our finding that 53.2% of root filled teeth were GQEW was better than the finding among the Taiwanese (30.3%) by Chueh et al., [27].

According to Chueh et al., [27], more cases treated in hospitals (38.1%) were GQEW than those from private dental clinics (24.3%). The difference in quality of endodontic work between hospitals and private clinics in Taiwan was very significant statistically (p≤0.001). Touré et al., [10] found that only 17.7% of root fillings in a Senegalese subpopulation were technically acceptable. In the Kosovar population, Kamberi et al., [29] found that following endodontic procedures by general dentists, 30.5% of root filled teeth had GQEW. While this is lower than our finding (53.2%), we believe that low standards and/or poor technique among general dentists could be responsible. Bjørndal et al., [30] evaluated the performance of general dental practitioners on factors they considered while performing root fillings. While the respondents emphasized factors associated with clinical symptoms, they under-rated the microbial situation of the tooth. We believe that improved undergraduate training, better equipment, higher professional standards and better technique would improve the quality of root canal fillings by general dentists. A developing economy like Nigeria would not have adequate number of endodontists for the population in the foreseeable future, hence reliance would continue to be on general dentists by large segments of the population.

On the basis of tooth location as anterior or posterior, Table 2 showed that among anterior teeth GQEW was done in 54.5% of teeth while the proportion in posterior teeth was lower at 34.5%. In Taiwan, Chueh et al., [27] found that GQEW was higher in anterior teeth (40.4%) than posterior teeth made up of premolars (33%) and molars (18.4%). This was much lower than the finding of Unal et al., [11] who analysed root fillings done by Turkish dental students on endodontics posting. The GQEW in anteriors were 90.1% while for posterior teeth it was 66.4%. Though statistical analysis revealed no significant difference in the quality of anterior and posterior teeth in our results, the technical difficulty of cleaning and effectively obturating multi-rooted (posterior) teeth at times with curved roots are well known. In the Turkish as well as this report, stainless steel files with step-back technique was used for all teeth. Kim et al., [31] recommends the use of nickel-titanium files and balanced force technique for curved roots as a way of increasing technical quality of fillings.

There are rare reports on the difference between the technical outcomes of endodontic treatment of maxillary or mandibular teeth. According to Kamberi et al., [29], the quality of endodontic work is directly associated with the likelihood of the development of apical periodontitis. However, while there is a 2:1 ratio of root filled teeth in the maxilla to the mandible, there is no difference in their average rate of apical periodontitis [29]. In Table 2, there were 47 maxillary root filled teeth (75.8%) while the rest were mandibular (n = 15, 24.2) giving a ratio of 3:1. There were almost equality in the quality of root fillings in upper teeth {GQEW was 26 (55.3%); NGQEW was 21 (44.7%)}. In the mandible, few teeth had GQEW (n = 3, 20%), the rest were NGQEW (80%) with statistical significance of p = 0.03. Possible reasons for the poor technical quality of mandibular root canal treatment are not known even though the small size of this sample is a limitation in assessing the clinical significance. Larger sized surveys would hopefully explain this issue further.

There has been controversy on the influence of coronal restoration in the outcome of root canal treatment and vice versa. Many authors reported that inadequate root fillings would jeopardize coronal restorations [32-35] but Ray & Trope [36] found that the quality of the coronal restoration had a greater impact on the periapical status of root canal treated teeth. This study is limited by the fact that periapical status and coronal restorations were not assessed, hence their relationship to coronal restorations could not be ascertained. However, it is impracticable to accurately predict the treatment outcome based on the quality of root canal fillings due to variations in patients’ health, teeth, biological, physical and psychological factors [37].

## Conclusion

The technical quality of root canal fillings has been variable from the literature due to technical and competency issues. As compared to earlier studies, this study showed that root fillings done by non- specialists had acceptable length of root canal fillings and homogeneity, while the overall quality was fair. The considerable experience in placement of root canal fillings gathered over the years by non specialist dental surgeons did not seem to improve their abilities as compared to their junior colleagues. The need for improved undergraduate and in-service training of non- specialists to improve their skills is stressed. There is general need for standardization of assessment criteria for technical outcome for international comparability. Worldwide, the quality of root canal fillings performed by general dentists need improvement for the benefit of patients.

## Competing interests

The authors declare that they have no competing interests.

## Authors’ contributions

ETA was the head of the study, did the radiographic reviews and made substantial contributions to conception and design of the study. LEA, RNN, ON, NAO and MOY participated in manuscript design and writing. NAH performed the statistical analysis. All authors read and approved the final manuscript.

## Pre-publication history

The pre-publication history for this paper can be accessed here:

http://www.biomedcentral.com/1472-6831/12/42/prepub
